# Is Follow-Up Endoscopy Necessary in Upper Gastrointestinal Cytomegalovirus Disease?

**DOI:** 10.1097/MD.0000000000003389

**Published:** 2016-05-13

**Authors:** Myeongsook Seo, Do Hoon Kim, Eun Jeong Gong, Ji Yong Ahn, Jeong Hoon Lee, Kee Wook Jung, Kee Don Choi, Ho June Song, Gin Hyug Lee, Hwoon-Yong Jung, Jin-Ho Kim, Sang-Oh Lee, Sang-Ho Choi, Yang Soo Kim, Jun Hee Woo, Sung-Han Kim

**Affiliations:** From the Department of Gastroenterology (MS, DHK, EJG, JYA, JHL, KWJ, KDC, HJS, GHL, H-YJ, J-HK) and Department of Infectious Diseases (S-OL, S-HC, YSK, JHW, S-HK), Asan Medical Center, University of Ulsan College of Medicine, Seoul, South Korea.

## Abstract

Gastrointestinal (GI) cytomegalovirus (CMV) disease is a major cause of morbidity and mortality in immunocompromised patients. Diagnosis of GI CMV disease mostly relies on endoscopy examination and histopathologic findings. There are limited data on the need for follow-up endoscopy with histopathologic examination in patients with upper gastrointestinal (UGI) CMV disease. All adult patients with confirmed and probable UGI CMV disease at a tertiary hospital over a 16-year period whose follow-up endoscopy was available were enrolled. The patients were classified as endoscopic responders if they showed complete or partial improvement on follow-up endoscopy, and as endoscopic nonresponders if there was no improvement or worsening. CMV tissue clearance was defined as absence of any visible CMV inclusion bodies, negative CMV immunohistochemistry and negative CMV polymerase chain reaction in follow-up biopsy tissues. During the study period, 77 patients with UGI CMV disease were analyzed. The median time to follow-up endoscopy was 19 days (interquartile range, 14–27). Of these 77 patients, 52 (68%) were classified as responders, and the remaining 25 (32%) as nonresponders. GI bleeding was more common in the nonresponders than the responders (36% vs 12%, respectively; *P* = 0.02). There was no significant difference in CMV tissue clearance between the responders and nonresponders (56% vs 69%, respectively; *P* = 0.38), median durations of treatment (20 days vs 21 days, respectively; *P* = 0.48), and relapse rates (10% vs 8%, respectively; *P* > 0.99). Multivariate analysis showed that the only independent predictive factor for relapse of CMV antigenemia or CMV GI disease was multiorgan CMV disease (odds ratio = 12.4, 95% confidence interval 1.6–97.9; *P* = 0.02). Endoscopic responses were obtained in about two-thirds of patients with UGI CMV disease 2 or 3 weeks after antiviral therapy. However, these follow-up endoscopic findings neither reflected CMV tissue clearance nor predicted disease relapse. These findings suggest that the routine follow-up endoscopy may not be warranted in patients with UGI CMV disease.

## Abstract 2

Supplemental Digital Content is available in the text

## INTRODUCTION

Gastrointestinal (GI) cytomegalovirus (CMV) disease is a major cause of morbidity and mortality in immunocompromised patients.^1^ Occasionally, reactivation of GI CMV disease also leads to severe complications in immunocompetent hosts, especially in the elderly.^2^ Diagnosis of GI CMV disease mostly relies on endoscopy examination with histopathologic findings. However, there are limited data on the need for follow-up endoscopy with histopathologic examination to document the absence of endoscopic or histologic evidence of persistent CMV disease before discontinuing antiviral therapy.^3^ To our knowledge, there is only 1 report showing that endoscopic resolution and tissue clearance were negatively associated with CMV relapse in 26 solid organ transplant recipients with CMV GI disease.^4^ However, the number of subjects was limited and only solid organ transplant recipients at high risk of CMV, namely those who were CMV D+/R− (donor seropositive and recipient seronegative), were evaluated. We therefore evaluated the need for follow-up endoscopy with histopathologic findings specifically in patients with upper gastrointestinal (UGI) CMV disease in whom follow-up endoscopy is easily performed.

## METHODS

### Study Population

We reviewed the medical records of patients diagnosed with confirmed and probable UGI CMV diseases between January 1999 and July 2014. The following parameters were collected: sex, age, underlying diseases, symptoms and signs at the time of diagnosis, involved sites of UGI tract, endoscopic findings, clinical outcome, and relapse. All patients diagnosed as UGI CMV disease received antiviral treatment, such as ganciclovir or valganciclovir. Antiviral therapy was administered at least until the resolution of clinical symptoms and CMV antigenemia. Follow-up endoscopy was performed at the discretion of the attending gastroenterologists. The CMV antigenemia assay was routinely performed in patients with UGI CMV disease. The CMV antignemia assay used the monoclonal antibodies C10/C11 (Biotest, Dreieich, Germany) and were performed as described elsewhere.^5^ Counts were expressed as the number of CMV antigen-positive cells per 200,000 leukocytes. This study was approved by the institutional review board of the Asan Medical Center, Seoul, South Korea (No. 2014-0780).

### Definitions

Confirmed UGI CMV disease was defined as detection of CMV by histology or immunohistochemical (IHC) staining with monoclonal antibody against CMV of biopsy specimens obtained by endoscopy, and presenting with signs or symptoms consistent with UGI disease. Probable CMV disease was defined as positive CMV polymerase chain reaction (PCR) from a biopsy specimen showing clinical improvement after antiviral therapy.^5^ The confirmed and probable UGI CMV patients in whom follow-up endoscopy was available were included in this study.

The patients were divided into 2 groups according to the endoscopic response in follow-up endoscopy, as described previously.^6^ The endoscopic responder group (RG) was defined as complete or partial improvement macroscopically on follow-up endoscopy. Complete improvement meant that mucosal healing was achieved in more than 90% of the affected area. Partial response indicated more than 50% mucosal healing. Absence of improvement or worsening on follow-up endoscopy was classified as endoscopic nonresponder group (non-RG). The endoscopic responses were determined by 2 experienced GI endoscopists (MS and DHK) in consensus. In addition, when an endoscopic response was unclear, cases were assessed by 2 other GI endoscopists (EJG and JYA), and the classification was reached by consensus. CMV tissue clearance was defined as absence of visible CMV inclusion bodies, negative CMV IHC staining and negative CMV PCR from follow-up biopsy tissue.

Immunocompromised patients were classified into the following groups: transplant recipients, namely those receiving liver, kidney, heart, pancreas, bone marrow, or multiple organs; patients with solid tumors receiving chemotherapy; those with human immunodeficiency virus (HIV) infections; those with hematologic malignancies; steroid users taking more than 30 mg prednisolone per day; patients with rheumatologic diseases undergoing immunosuppressive therapy.^2^ Involved sites of the UGI tract were classified as esophagus, stomach, or duodenum. Instances of multiple sites of GI infections consisted of 1 case each involving esophagus and stomach, stomach and duodenum, and esophagus and duodenum. Simultaneous involvement of both upper and lower GI tract was classified as extensive GI CMV disease. Multiorgan CMV disease was defined as involvement of 2 or more organs, usually a combination of lung, retina, and GI tract. GI bleeding is diagnosed by the presence of hematemesis, melena, hematochezia, or blood in a patient's gastric contents on lavage. Relapse after antiviral treatment was to be CMV antigenemia or a CMV GI disease such as esophagitis, gastritis, and colitis.

### Statistical Analysis

Continuous variables were expressed as means with standard deviations or medians with interquartile ranges (IQRs), as appropriate. For comparisons of baseline and clinicopathologic characteristics in the RG and non-RG, continuous parameters were analyzed by Student *t* test or the Mann–Whitney *U* test, and categorical variables were compared using the χ^2^ test or Fisher exact test, as appropriate. The Kaplan–Meier method was used to construct survival curves for the percentages of patients with CMV clearance from tissue and negative CMV antigenemia. The log-rank test was used to compare time to CMV clearance between endoscopic responders and nonresponders. Times to CMV loss of antigenemia and tissue clearance were compared using the Wilcoxon signed-rank test. Independent predictors of relapse of CMV antigenemia or CMV GI disease, and of all-cause in-hospital mortality were identified by means of forward stepwise logistic regression analyses of factors with *P* ≤ 0.05 in univariate analysis. Values of *P* ≤ 0.05 were considered statistically significant. All statistical analyses were performed with SPSS version 21 for Windows (SPSS, Inc., Chicago, IL).

## RESULTS

### Baseline and Clinical Characteristics

**FIGURE 1:**
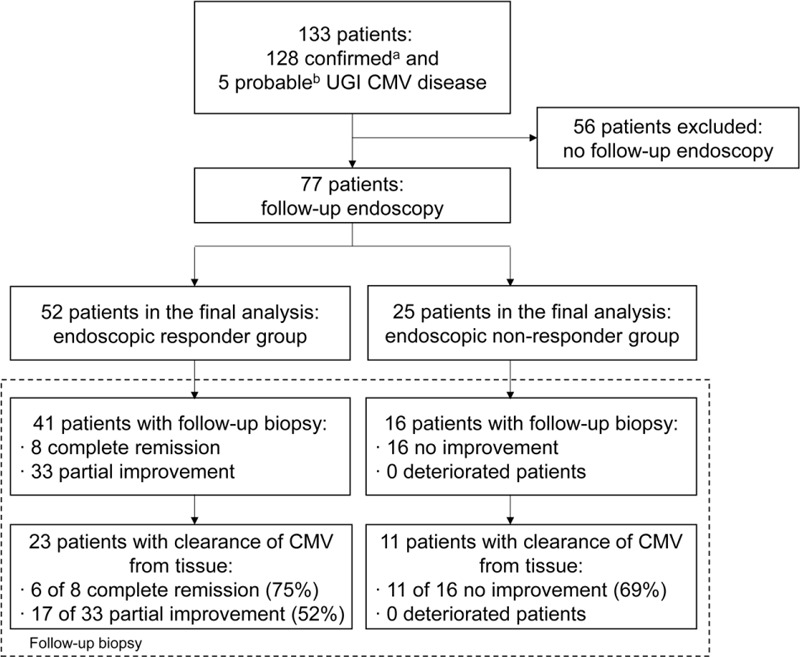
Flow chart of patients. CMV = cytomegalovirus, UGI = upper gastrointestinal. ^a^Confirmed UGI CMV disease was defined as symptoms or signs of gastrointestinal disease with positive pathologic findings of CMV infection from hematoxylin and eosin (H&E) staining and immunochemical staining of endoscopic biopsies. ^b^Probable disease was defined as a positive CMV polymerase chain reaction from a biopsy specimen showing clinical improvement with antiviral therapy.

Of the total of 133 patients including 128 confirmed and 5 probable UGI CMV cases, 77 (58%) underwent follow-up endoscopy (Figure 1). These 77 patients were analyzed in this study. The clinical characteristics and outcomes of these (n = 77) patients who underwent follow-up endoscopy and those (n = 56) who did not are shown in Supplemental Table 1. Of the 77 patients, 52 (68%) were classified as responders (RG) and the remaining 25 (32%) as nonresponders (non-RG). Demographic and clinical characteristics are shown in Table 1. The RG showed a tendency to include a greater proportion of transplant recipients than the non-RG (67% vs 44%, respectively; *P* = 0.051). However, GI bleeding was more common in the non-RG than the RG (36% vs 12%; *P* = 0.02). The median level (IQR) of CMV antigenemia did not differ significantly between the 2 groups (62 [IQR, 6–361] in the RG vs 107 [IQR, 3–454] in the non-RG; *P* = 0.96). There was no significant difference between both groups in regard to steroid, nonsteroidal anti-inflammatory drug, antiplatelet agent, and warfarin, which could increase the risk of GI bleeding before the diagnosis of CMV diseases. The rates of proton pump inhibitor (PPI) use were not different between 2 groups before the diagnosis of CMV disease. However, PPI was more frequently used in the non-RG than the RG (92% vs 69%; *P* = 0.03).

**TABLE 1:**
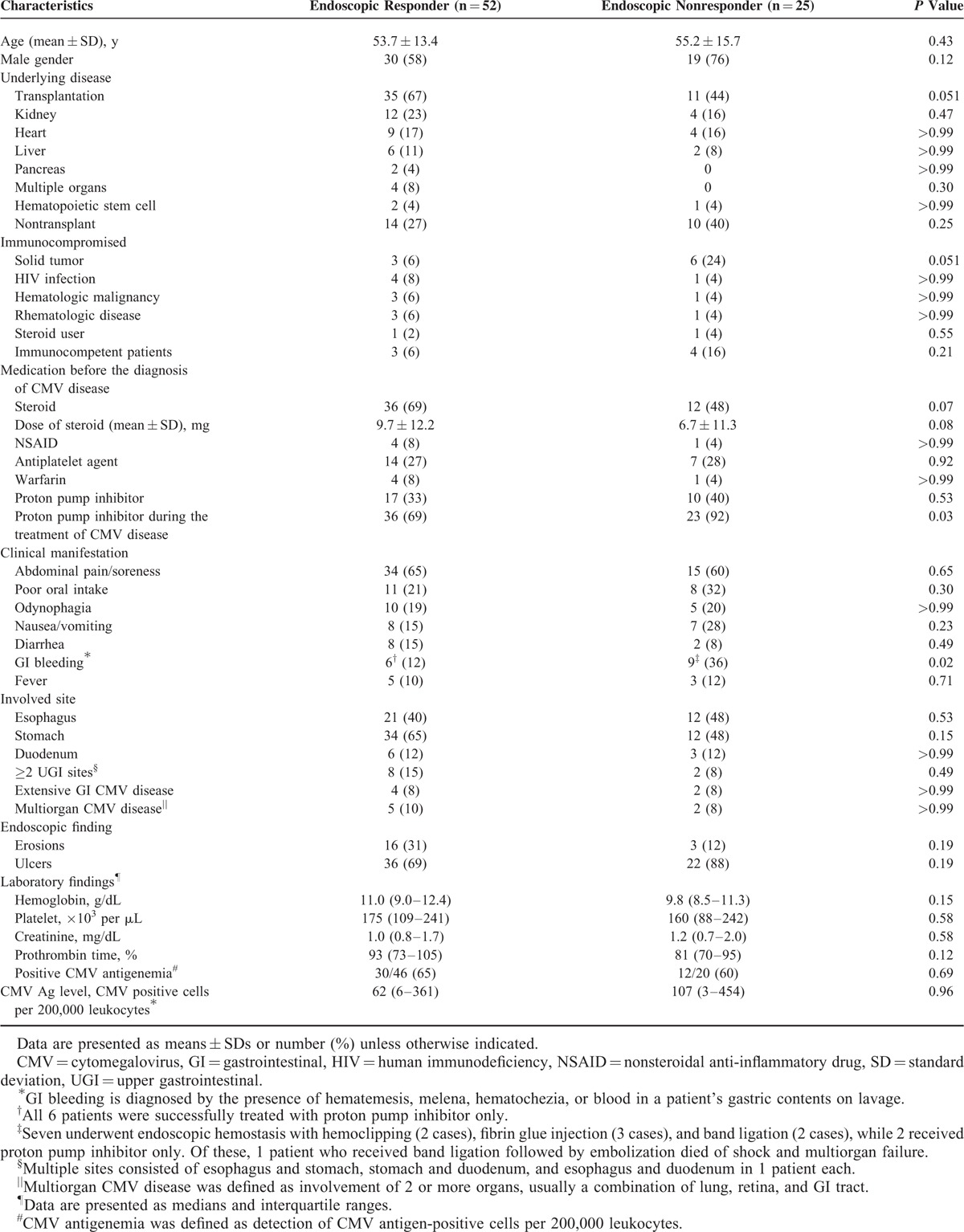
Clinical Characteristics of Patients According to Endoscopic Response

### Comparison of Viral Clearance and Clinical Outcomes

**FIGURE 2:**
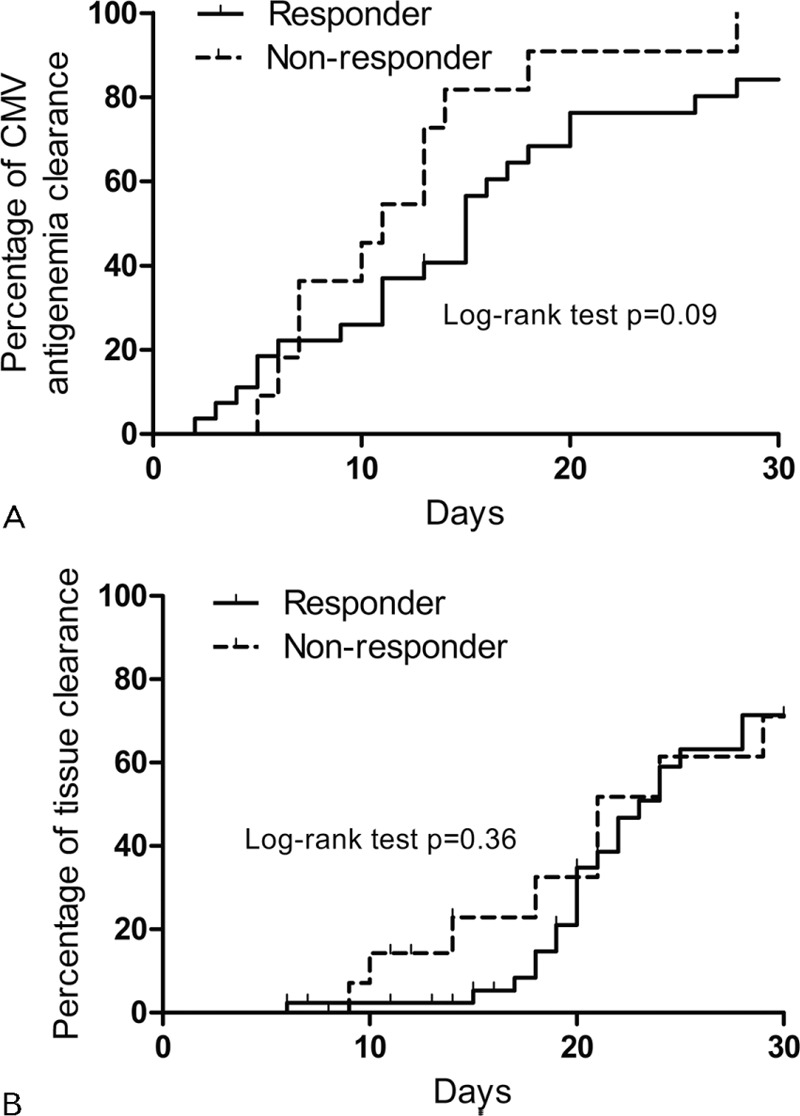
Kaplan–Meier curves of cumulative percentages of viral clearance from blood (A) and tissue (B) in endoscopic responders and nonresponders. The time to blood (A) and tissue (B) clearance did not differ between the 2 groups.

Antiviral therapy such as ganciclovir or valganciclovir was used in all cases. The clinical and histological outcomes are shown in Table 2. There was no significant difference in the median time to CMV antigenemia clearance during antiviral treatment between the 2 groups (15 days [IQR, 8–20] in the RG vs 11 days [IQR, 7–14] in the non-RG; *P* = 0.31) (Figure 2A; log-rank test, *P* = 0.09). The median time to follow-up endoscopy was shorter in the non-RG than the RG (14 days [IQR 8–21] vs 20 days [IQR 15–29]; *P* = 0.002). A follow-up biopsy was performed in 41 (79%) of the RG and 16 (64%) of the non-RG. The clinical characteristics and outcomes of those (n = 57) who underwent follow-up biopsy and those (n = 20) who did not are shown in Supplemental Table 2. There was no significant difference in CMV tissue clearance between the RG and non-RG (56% vs 69%, *P* = 0.38); CMV tissue clearance was observed in 6 (75%) of 8 patients with complete remission, 17 (52%) of 33 patients with partial improvement, and 11 (69%) of 16 patients with worsening disease. There was also no significant difference in the median time to tissue clearance between the RG (23 days [IQR 19–28]) and the non-RG (21 days [IQR 14–29], *P* = 0.11) (Figure 2B; log-rank test, *P* = 0.36). Overall, CMV clearance from tissue (median 20 days [IQR 17–23]) took a longer time than from blood (median 13 days [IQR 7–18], *P* = 0.03).

**TABLE 2:**
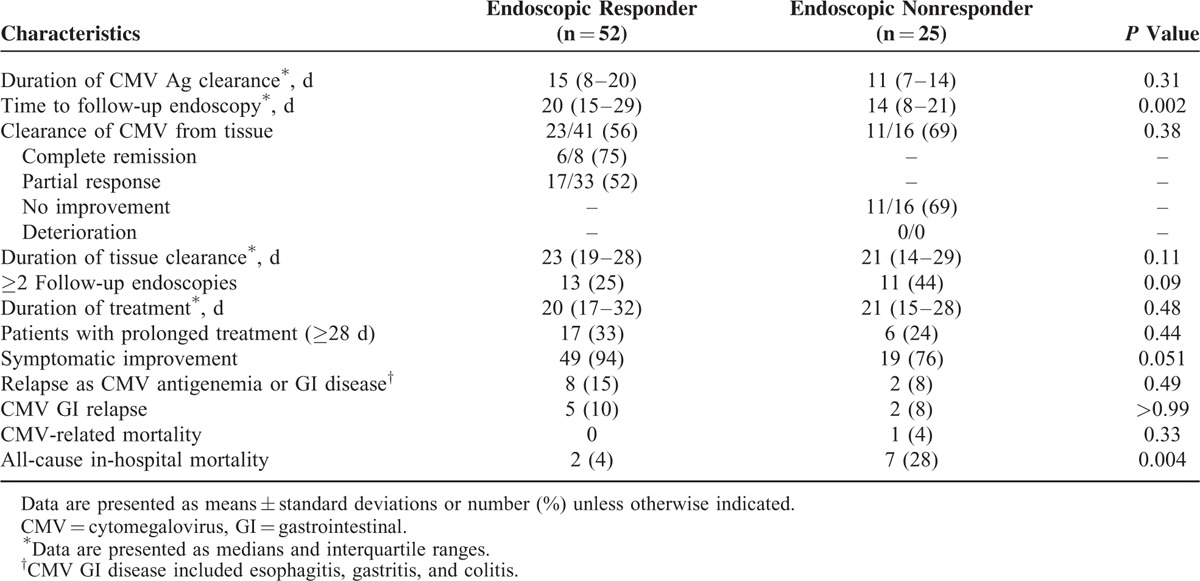
Clinical Outcomes in Endoscopic Responders and Nonresponders

The median duration of treatment (20 days vs 21 days, respectively; *P* = 0.48) was similar in the 2 groups. In addition, the proportion of patients who received treatment for more than 4 weeks did not differ significantly (17 [33%] in the RG vs 6 [24%] in the non-RG, *P* = 0.44). There was a trend toward higher symptomatic improvement in the RG (49/52 [94%]) than in the non-RG (19/25 [76%], *P* = 0.051).

### Relapse Rates and Risk Factors for CMV Relapse and Mortality

Recurrence of CMV antigenemia and CMV GI disease was similar in the RG (8/52 [15%]) and non-RG (2/25 [8%]) (*P* = 0.49). The CMV GI relapse rate was also similar (RG (5/52 [10%]) and non-RG (2/25 [8%]) (*P* > 0.99). The analysis of risk factors for relapse of CMV antigenemia and CMV GI disease are shown in Table 3. Univariate analysis revealed that multiorgan CMV disease was associated with relapse of CMV (*P* = 0.04). However, transplant recipient, endoscopic ulceration, high level of CMV antigenemia, endoscopic nonresponders, nonclearance of CMV from tissue, delayed CMV antigenemia clearance (≥14 days), and prolonged treatment were not risk factors for CMV relapse. Multivariate analysis showed that the only independent predictive factor for relapse of CMV antigenemia or CMV GI disease was multiorgan CMV disease (odds ratio [OR] = 12.4, 95% confidence interval [CI] 1.6–97.9; *P* = 0.02).

**TABLE 3:**
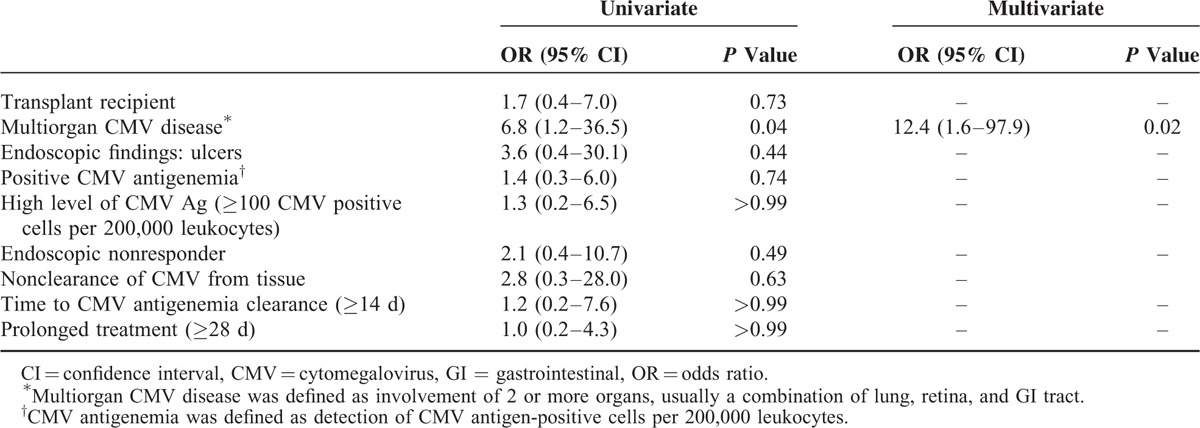
Univariate and Multivariate Analyses of Risk Factors for Recurrence of CMV Antigenemia or CMV GI Disease

However, the all-cause in-hospital mortality rate was higher in the non-RG (7/25 [28%]) than in the RG (2/52 [4%]) (*P* = 0.004). An analysis of predictive factors for all-cause in-hospital mortality is shown in Table 4. Univariate analysis demonstrated that the predictive factors for all-cause in-hospital mortality were GI bleeding, nongastric involvement, endoscopic nonresponder, mechanical ventilation, anemia (hemoglobin ≤9.0 g/dL), and thrombocytopenia (≤100,000/μL). In multivariate analysis, the only independent predictive factor for all-cause in-hospital mortality was GI bleeding (OR = 11.0, 95% CI 2.2–56.2; *P* = 0.004).

**TABLE 4:**
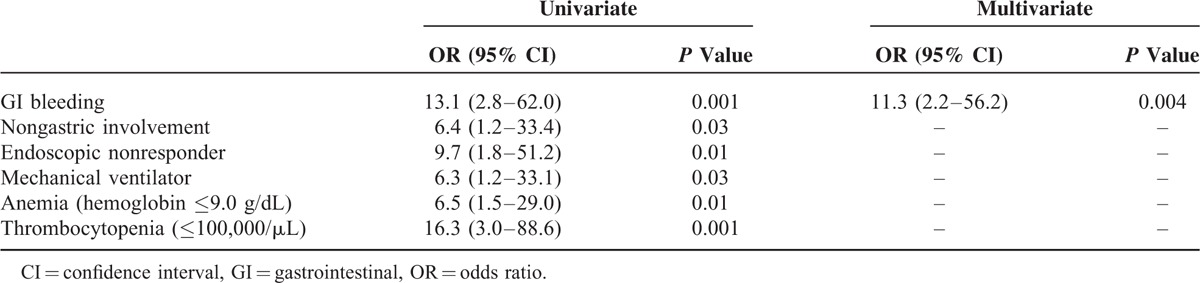
Univariate and Multivariate Analyses of the Predictive Factors for All-Cause In-Hospital Mortality

## DISCUSSION

We found no significant differences between the endoscopic responders and nonresponders in terms of viral tissue clearance rate and relapse rate. Therefore, the follow-up endoscopic findings did not reflect CMV tissue clearance or predict disease relapse. Our study thus suggests that routine follow-up endoscopy may not be warranted in patients with UGI CMV disease.

Previous studies showed that 23% to 39% of CMV disease recurred after antiviral treatment,^7–9^ In addition, Sia et al^7^ and Humar et al^8^ observed relapse rates of CMV viremia and CMV disease of 33% (8/24) and 23% (12/52), respectively, in solid organ transplant recipients, and relapse rates of CMV disease of 1% (1/8) and 21% (3/14), respectively. Wilcox et al^6^ also found that the relapse rate of CMV esophagitis in HIV-infected patients was 39% (7/18). In contrast, this study revealed a relapse rate of CMV antigenmia or CMV GI disease of 13% (10/77) in patients with CMV UGI disease. In addition, the CMV GI relapse rate was 9% (7/77). Previous studies have generally dealt with HIV-infected patients^6^ or solid organ transplant recipients who are at a high-risk CMV, such as CMV D+/R− (donor seropositive and recipient seronegative) cases.^7,8^ However, one-third of the patients in our study were nontransplant patients, including 5 HIV-infected patients. Furthermore, since more than 95% of Korean adults are seropositive for CMV IgG,^9^ the transplant recipients were mostly CMV R+ (recipients seropositive). This partially explains why the CMV relapse rate in our study was lower than that in previous studies.

The median time to CMV antigenemia clearance was similar in the endoscopic responders and nonresponders as was the tissue CMV clearance rate. Therefore, endoscopic response did not guarantee tissue clearance from the UGI tract. However, CMV tissue clearance (median 20 days) took longer than clearance from the blood (median 13 days, *P* = 0.03). This suggests that the clearance of CMV from locally inflamed sites after antiviral therapy may be delayed compared with clearance from the blood, in line with a previous study.^4^

Eid et al have shown that endoscopic resolution of GI disease and viral clearance from the GI tract were not protective factors for CMV relapse. Only extensive GI disease with involvement of upper and lower GI tracts was significantly associated with a higher risk of CMV relapse.^4^ Falagas et al^10^ found that multiorgan CMV disease was independently associated with recurrent CMV disease in orthotopic liver transplant recipients. Our data showed that CMV relapse was not associated with viral clearance from blood or tissue or with gross endoscopic response. Multiorgan CMV disease was a predictive factor for CMV relapse in univariate and multivariate analyses. CMV infections are frequently caused by CMV reactivation, which is associated with the loss of CMV-specific T-cell immunity. The recently developed enzyme-linked immunospot assay quantifies T cells producing interferon-γ in response to CMV and can predict CMV disease and viremia.^11,12^ Hence we assume that CMV relapse may be related to CMV-specific immunity. Further studies are needed to define the risk factors or predictive factors for relapse of CMV.

Our study had a few limitations. First, it had a retrospective design. Also the time interval to follow-up endoscopy was not fixed and endoscopy was performed at the discretion of each attending physician. Therefore, a well-designed prospective cohort study with protocol-based follow-up endoscopy could provide more valuable information on the necessity for follow-up endoscopy. Nonetheless, we believe that this is the largest study to date to assess the need for follow-up endoscopy for confirming tissue clearance of CMV. So, until prospective clinical trials are available on this issue, alternative study designs, such as one used in our study, are often needed to answer important policy questions. Second, there may have been a selection bias because mild cases that did not undergo follow-up endoscopy might have been excluded. However, Supplemental Table 1, shows that there were no differences in underlying disease, involved sites, endoscopic findings, and proportion of positive CMV antigenemia between the patients who did and those who did not undergo follow-up endoscopy. In addition, the median duration of CMV antigenemia clearance, rates of symptomatic responders, and mortality (including all-cause in-hospital and CMV-related mortality) and relapse were similar except for the median duration of antiviral treatment. Therefore, our results suggest that this type of selection bias did not affect our study results substantially. Finally, it is difficult to extrapolate our results to CMV disease in the entire or lower GI since our study included only upper GI CMV disease.

In conclusion, endoscopic responses were seen in about two-thirds of patients with UGI CMV disease after 2 or 3 weeks of antiviral therapy. However, follow-up endoscopic findings did not reflect CMV tissue clearance or predict disease relapse. These observations suggest that routine follow-up endoscopy may not be warranted in patients with UGI CMV disease.

## Supplementary Material

Supplemental Digital Content
