# Supplementary material for: Is Follow-Up Endoscopy Necessary in Upper Gastrointestinal Cytomegalovirus Disease?
Source: Medicine (Baltimore). 2016 May 13;95(19):e3389. doi: 10.1097/MD.0000000000003389 (PMC4902479; doi:10.1097/MD.0000000000003389)
Supplement: Supplemental Digital Content [file medi-95-e3389-s001.doc]

**SUPPLEMENTAL TABLE 1. Comparison of Clinical Characteristics and Outcomes in Patients Who Did and Did Not Undergo Follow-up Endoscopy**

| **Characteristic** | **Follow-up endoscopy (n = 77)** | **No follow-up**  **endoscopy (n = 56)** | ***P* value** |
| --- | --- | --- | --- |
| **Age (mean ± SD), years** | 54.2 ± 14.1 | 55.02 ± 15.4 | 0.75 |
| **Male gender** | 49 (64) | 33 (59) | 0.58 |
| **Underlying disease**  Transplantation  Non-transplant patients | 46 (60)  31 (60) | 36 (64)  20 (36) | 0.60  0.25 |
| **Involved site**  Esophagus  Stomach  Duodenum  ≥2 UGI sites^†^  Extensive GI CMV disease  Multiorgan CMV disease^‡^ | 33 (43)  46 (60)  9 (12)  10 (13)  7 (9)  7 (9) | 22 (39)  38 (68)  2 (4)  5 (9)  6 (11)  4 (7) | 0.68  0.34  0.12  0.47  0.76  0.76 |
| **Endoscopic finding**  Erosions  Ulcers | 57 (74)  15 (21) | 41 (73)  10 (18) | 0.92  0.68 |
| **Positive CMV antigenemia**^§^ | 42/66 (64) | 40/51 (78) | 0.08 |
| **CMV Ag level,** CMV positive cells/200,000 leukocytes^*^ | 90 (6-361) | 46 (9-248) | 0.95 |
| **Time to CMV Ag clearance**, days^*^ | 13 (7-18) | 14 (10-20) | 0.67 |
| **Duration of treatment**, days^*^ | 21 (17-28) | 15 (13-23) | 0.001 |
| **Patients with prolonged treatment (≥28 days)** | 23 (30) | 8 (14) | 0.036 |
| **Symptomatic improvement** | 68(88) | 47 (84) | 0.47 |
| **Relapse as CMV antigenemia or GI disease** | 10 (13) | 3 (5) | 0.14 |
| **CMV GI relapse** | 7 (9) | 2 (4) | 0.30 |
| **CMV-related mortality** | 1 (1) | 0 | >0.99 |
| **All-cause in-hospital mortality** | 9 (12) | 11 (20) | 0.21 |

SD = standard deviation, GI = gastrointestinal, CMV = cytomegalovirus.

**NOTE.** Data are presented as mean ± SD or number (%) unless otherwise indicated.

^*^Data are presented as medians and interquartile ranges (IQR).

^†^Multiple sites included the esophagus and stomach, stomach and duodenum, and esophagus and duodenum in one patient each.

^‡^Multiorgan CMV disease was defined as involvement of two or more organs, usually a combination of lung, retina and GI tract.

^§^CMV antigenemia was defined as detection of CMV antigen-positive cells per 200,000 leukocytes.

**SUPPLEMENTAL TABLE 2. Comparison of Clinical Characteristics and Outcomes Between Patients Who Did and Did Not Undergo Follow-up Endoscopic B**iopsy

| **Characteristic** | **Follow-up biopsy**  **(n = 57)** | **Without follow-up biopsy**  **(n = 20)** | ***P* value** |
| --- | --- | --- | --- |
| **Age (mean ± SD), years** | 55.4 ± 12.6 | 50.7 ± 17.6 | 0.49 |
| **Male gender** | 34 (60) | 15 (75) | 0.22 |
| **Underlying disease**  Transplantation  Non-transplant patients | 36 (63)  21 (37) | 10 (50)  10 (50) | 0.30  0.30 |
| **Involved site**  Esophagus  Stomach  Duodenum  ≥ 2 UGI sites^†^  Extensive GI CMV disease  Multiorgan CMV disease^‡^ | 21 (37)  38 (67)  8 (14)  9 (16)  5 (9)  5 (9) | 12 (60)  8 (40)  1 (5)  1 (5)  1 (5)  4 (7) | 0.07  0.04  0.43  0.44  >0.99  >0.99 |
| **Endoscopic finding**  Erosions  Ulcers | 13 (23)  41 (72) | 3 (15)  16 (80) | 0.54  0.57 |
| **Positive CMV antigenemia**^§^ | 33/50 (66) | 9/16 (56) | 0.48 |
| **CMV Ag level,** CMV positive cells/200,000 leukocytes^*^ | 90 (6-361) | 46 (9-248) | 0.87 |
| **Duration of CMV Ag clearance**, days^*^ | 13 (7-18) | 14 (10-21) | 0.42 |
| **Endoscopic responder** | 41 (72) | 11 (55) | 0.16 |
| **Duration of treatment**, days^*^ | 21 (17-28) | 15 (13-23) | 0.28 |
| **Patients with prolonged treatment (≥28 days)** | 17 (29) | 6 (30) | >0.99 |
| **Symptomatic improvement** | 52 (91) | 16 (80) | 0.23 |
| **Relapse as CMV antigenemia or GI disease** | 9 (16) | 1 (5) | 0.44 |
| **CMV GI relapse** | 6 (11) | 1 (5) | 0.67 |
| **CMV-related mortality** | 0 | 1 (5) | 0.26 |
| **All-cause in-hospital mortality** | 6 (11) | 3 (15) | 0.69 |

SD = standard deviation, GI = gastrointestinal, CMV = cytomegalovirus.

**NOTE.** Data are presented as means±SD or numbers (%) unless otherwise indicated.

^*^Data are presented as medians and interquartile ranges (IQR).

^†^Multiple sites included esophagus and stomach, stomach and duodenum, and esophagus and duodenum in one patient each.

^‡^Multiorgan CMV disease was defined as involvement of two or more organs, usually a combination of lung, retina and GI tract.

^§^CMV antigenemia was defined as detection of CMV antigen-positive cells per 200,000 leukocytes.
